# Prevalence of *Toxoplasma gondii* Measured by Western Blot, ELISA and DNA Analysis, by PCR, in Cats of Western Mexico

**DOI:** 10.3390/pathogens11010109

**Published:** 2022-01-17

**Authors:** María de la Luz Galván-Ramírez, Claudia Charles-Niño, César Pedroza-Roldán, Carolina Salazar-Reveles, Karen Lissete Ocampo-Figueroa, Laura Roció Rodríguez-Pérez, Varinia Margarita Paez-Magallán

**Affiliations:** 1 Departamento de Microbiología y Patología, Centro Universitario de Ciencias de la Salud, Universidad de Guadalajara, Guadalajara C.P. 44340, Mexico; rocio2427@hotmail.com; 2 Departamento de Medicina Veterinaria, Centro Universitario de Ciencias Biologicas y Agropecuarias, Universidad de Guadalajara, Zapopan C.P. 45187, Mexico; srita.1288@gmail.com (C.S.-R.); ocampo2211@gmail.com (K.L.O.-F.); variniapm@hotmail.com (V.M.P.-M.)

**Keywords:** toxoplasmosis, cats, serology, western Mexico, molecular detection, western blot

## Abstract

*Toxoplasma gondii* is the causative agent of toxoplasmosis in humans and animals. The sexual reproductive cycle of *Toxoplasma* takes place in the small intestine of felines, the definitive hosts. In the final part of the sexual cycle, *T. gondii* forms oocysts in infected cats. Oocysts transferred via the faeces to the environment are highly infectious to both animals and humans. This study aimed to determine the prevalence and risk factors associated with *T. gondii* infection in cats from the metropolitan region of Guadalajara in western Mexico. Western blotting and ELISA for anti-*Toxoplasma* IgG antibodies was performed, and *Toxoplasma* DNA was identified using polymerase chain reaction. Prevalence of anti-*T. gondii* antibodies was 14.8% (44/297), and only 2/297 cases were positive for PCR. Cats older than one year were at an increased risk of infection (OR = 3.9, 95% CI 1.844–8.362). Sex, raw meat feeding, hunting habits, vaccination status, and body condition were not associated with positivity. The prevalence of *T. gondii* infection determined with Western blot in cats in the metropolitan area of Guadalajara, Jalisco, Mexico, was lower than that reported in previous studies.

## 1. Introduction

*Toxoplasma gondii* is an intracellular parasite that presents a significant threat to public health. Congenital toxoplasmosis occurs when the mother is first infected with *Toxoplasma* during pregnancy; the parasite can infect the foetus, causing death or severe neurological impairment, inflammation, and retinochoroiditis [1,2]. Immunocompromised patients are associated with severe damage to the central nervous system, lethal encephalitis, and myocarditis [1,2]. Infection can be acquired by various mechanisms: vertical transmission, from mother to child; orally, via cysts present in raw or undercooked meat [1]; or oocysts present in water and/or fruit and vegetables watered with sewage water and eaten without washing [2]. Other mechanisms include infected organ transplants, blood transfusions, and direct contamination when working in laboratories with hand wounds when the parasite or contaminated raw meat is handled. 

The reproductive sexual cycle of *Toxoplasma* takes place only in the definitive hosts (domestic and wild cats). Gastric enzymes destroy the cyst wall in the small intestine after ingestion of the cysts present in the host tissues. Approximately 15 to 20 days after infection, cats shed more than 100 million oocysts in their faeces [3]. Moreover, oocysts are highly resistant to adverse environmental conditions, increasing the risk of infection in humans and animals [4,5]. *Toxoplasma* infection in cats is, in most cases, asymptomatic, complicating the diagnosis of the disease by veterinarians. 

The epidemiology in humans in Mexico in a study of the meta-analysis of 41 publications and 70,123 individuals showed the average mean weighted prevalence was 27.97%. The prevalence was higher in women with miscarriages (36.03%), immunocompromised patients (28.54%), mentally ill patients (38.52%), and other risk groups (35.13%). However, *Toxoplasma* infection among the Mexican population showed a downward trend of 0.1%/year over a period of sixty years, which represents a 5.8% reduction in prevalence [6].

Epidemiological reports of Latin American countries have shown a high prevalence of *Toxoplasma* infection in cats (41.9%) [7,8,9]. Similarly, in Mexico, epidemiological surveys conducted in cats in the last ten years have indicated an average prevalence of 40.8%. States such as Colima, Durango, and Mexico City have reported prevalence rates of 28.8%, 21%, and 21.8%, respectively [10,11,12]. In contrast, in the Yucatan, a high prevalence of 91.8% of cats has been reported [7]. In Jalisco, a study in 1999 identified a prevalence of 70.8% for IgG and 8.3% for IgM anti-*Toxoplasma* antibodies [13]. In the last ten years, Mexican federal and local regulations, promoted by the Mexican Association of Veterinarians Specializing in Small Species (AMMVEPE), have increased the number of shelters, providing further support to stray cats and facilitating adoption.

In the metropolitan area of Guadalajara, seroprevalence studies associated with *Toxoplasma gondii* infection in cats have not been performed for more than 20 years. Therefore, the seroprevalence of 70% reported by Galvan et al. in 1999 was used as the statistical data as a reference [13]. There is no precise census of the cat population; however, according to the Mexican Association of Veterinarians Specializing in Small Species (AMMVEPE), the region contains 23 million dogs and cats, of which 6,900,000 are pets, while the rest live on the street. Of these 6 million, 2,070,000 were estimated to be cats. Specifically, in the Guadalajara metropolitan area, there were 73,000 cats.

There is no existing cat census in the metropolitan region of Guadalajara; however, in 2016, it was estimated that the region homed 100,000 cats, of which 25% were strays [14]. The objective of this study was thus to determine the prevalence of *T. gondii* antibodies and DNA in cats and to describe the possible associated risk factors. 

## 2. Results

### 2.1. Questionnaire

Of the 44 cats, 19 (6.3%) were shelter cats and 25 (8.4%) were home cats; the statistical analysis between both categories showed no significant statistical difference. Of the households where the sampling was carried out, 15% of them had more than four cats, of which at least two were positive.

The average age of the cats included in this study was 20.7 ± 27.8 months, with a significant standard deviation due to the age dispersion, which varied from 1 month to 156 months (13 years of age for the oldest cat). The positivity of anti-*Toxoplasma* antibodies was analysed through Western blot in the two age groups: <one year, 7.8% positive; ≥one year, 24%. A statistically significant difference (*p* < 0.001) was found between the two age groups. 

The prevalence of anti-*Toxoplasma* antibodies in cats was further analysed by sex; the female group showed a slightly higher positivity rate than males (16.3 vs. 13.6%, respectively). Analysis between groups was performed using the chi-square test, or Student’s t-test for independent samples and did not reveal any statistically significant differences (Table 1).

**Figure 1:**
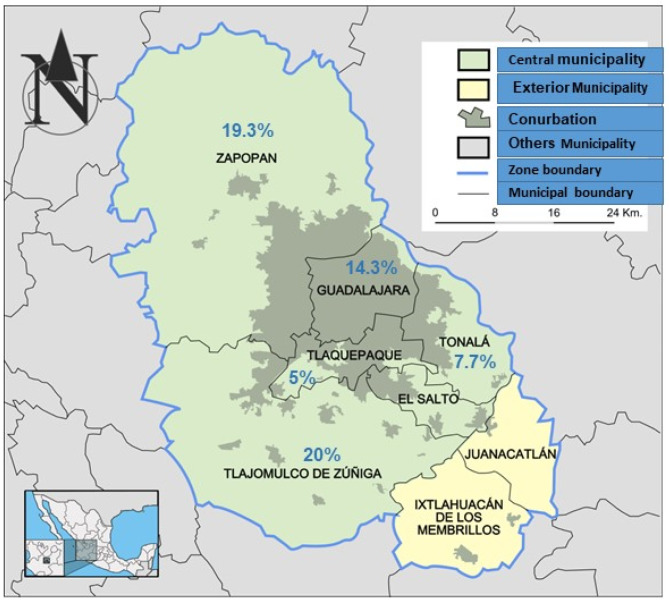
Map showing the geographic distribution of the frequencies (%) of positivity of *Toxoplasma* antibodies found in five municipalities of the metropolitan zone of Guadalajara, Jalisco, Mexico. The frequency of infected cats was higher in cities with a high population density per km^2^: Zapopan has 9721.36 inhabitants, Tlajomulco de Zúñiga 2459.97, and Guadalajara 1491.57.

The highest prevalence of anti-*Toxoplasma* antibodies was observed in the municipalities of Tlajomulco de Zuñiga 20%, Zapopan 19.6%, and 14.3% Guadalajara. Tonalá and San Pedro Tlaquepaque had lower seroprevalence rates of 7.7 and 5%, respectively (Table 1). According to municipalities in the metropolitan zone of Guadalajara, the geographic distribution of positive cats was higher in cities with a high population density per km^2^, as shown in Figure 1 and Table 1. 

### 2.2. Lifestyle and Risk Factors of the Cats Studied 

Positivity to anti-*T. gondii* IgG antibodies was analysed in the presence or absence of the following variables: complete vaccination, consumed raw meat, other animals in the same habitat, current deworming, and body condition. However, we did not find any statistically significant differences in any of the variables Table 2. 

Cats positive to PCR, western blot and ELISA.

The cat 21/10-222 was male and less than 2 years old. It was identified in the municipality of Guadalajara but was originally adopted from the state of Sonora, Mexico. The cat was vaccinated with the triple feline vaccine and had been dewormed within the last 6 months. The cat’s staple food was kibble in combination with raw meat on a few occasions. The cat did not present hunting habits according to the owners. 

On the other hand, cat 24/10-223 was male and less than 4 years old and was identified in the municipality of Guadalajara. The owners stated that the cat was not vaccinated or dewormed. The owners also confirmed that the cat had hunting habits and had captured insects and small birds on several occasions. During the general physical examination of this cat, fleas were identified, and an external and internal deworming treatment was prescribed.

### 2.3. Western Blot 

**Figure 2:**
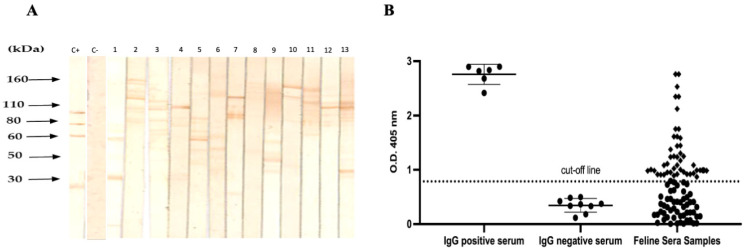
Reactivity against *T. gondii* lysates of serum samples obtained from cats by Western blotting and ELISA. (**A**) A representative WB of the reactivity of the serum against antigens of *T. gondii* shown, a pattern of bands with a range of 27 to 130 kDa. C + positive control serum and C-feline negative control serum. (**B**) Western blotting revealed 44 positive and 40 negative samples, which were subsequently analysed by ELISA immunoassay. The mean 0.75 ± 0.034 standard deviations of optical density (O.D.) from negative controls were used to generate the cut-off line.

The frequency of IgG anti-*Toxoplasma* antibodies was determined by Western blotting in 297 cats. The prevalence was lower than expected; 44/297 (14.81%) samples were positive while 253/297 were negative. The positive samples showed different band patterns corresponding to the molecular weights of tachyzoite proteins in the range of 27 to 120 kDa. However, most of the positive serum samples produced bands in the range of 60–120 kDa, and, finally, no bands were identified in the serum of normal cats (Figure 2A). 

### 2.4. Immunoassay ELISA 

A home ELISA immunoassay confirmed the positive serum samples in Western blotting of 44 positives and 40 negatives. The optical densities of positive and negative samples were compared, establishing the presence of specific IgG antibodies against *T. gondii* antigens in these samples (Figure 2B).

### 2.5. Detection of T. gondii DNA

**Figure 3:**
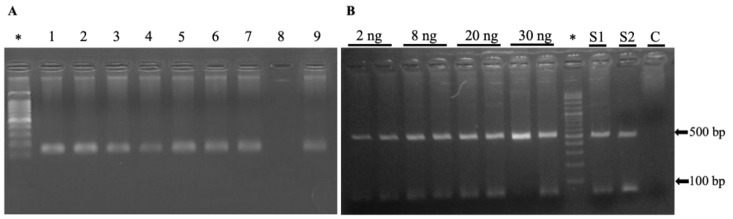
Molecular identification of *Toxoplasma gondii*. (**A**) Genomic DNA extracted from the blood of cats was subjected to PCR amplification targeting a 120 bp fragment of the β-actin gene as a positive control to validate the integrity of the extracted DNA. Lanes show the result of nine samples; only the sample in line 8 was discarded from the study for the absence of the product. (**B**) Those samples positive for β-actin were subjected to diagnosis by the presence or absence of the *B*1 gene associated with *T. gondii* (approximately 470 bp according to bioinformatics analysis). The first lines of the agarose gel (2%) show the sensitivity of PCR using different concentrations (2 to 30 ng) of *T. gondii* genomic DNA as a positive control. Line S1 and S2 show positive amplification to *T. gondii* from two cats’ genomic DNA, and line C shows the negative internal control. Lines marked with * show 100 bp DNA ladder.

Detection of *T. gondii* DNA in the blood of the 297 samples was performed via a nested polymerase chain reaction; only 2/297 were positive, representing 0.7% of recent infections in the studied cats (Figure 3). Confirmation by DNA sequencing of the B1 gene of *Toxoplasma gondii*. See in Supplementary File S1.

## 3. Discussion

In our study, PCR analysis of the *Toxoplasma* B1 gene was found only in two samples (0.7%) that were positive for B1. However, this result is not without precedent; in a study in Korea, 1312 samples of cat blood were analysed using the same B1 gene with PCR method, identifying only five positive samples [15]. The two samples positive by PCR were also positive by Western blot; this is possibly due to tachyzoites in blood by acute infection and low sensitivity of PCR [15,16]. 

The results found in this study showed a low percentage (14.8%) of IgG antibodies determined by Western blotting and tested through ELISA. The Western blot shows patterns of *T. gondii* infection were heterogeneous, in the range of 30–120 kDa [17,18]. The recognition of *T. gondii* antigens by cat IgG antibodies may be different for several reasons, including the type of antigen (tachyzoites, bradyzoite/cysts, and oocyst/sporozoites), the secondary antibody used (polyclonal or monoclonal [19]), strain, guests, and time of infection. This aspect has been demonstrated in cats experimentally infected with the *Toxoplasma* strain ME49 [20]. These results are similar to those of another study performed in Korea regarding the different proteins recognised by IgG antibodies in infected cats. This study used Western blotting as a reference test and was confirmed with ELISA and found that cat serum tested positive in both methods; our results were further compared by ELISA. This suggests the high sensitivity and specificity of Western blotting [19]. In Russia, a kit for the detection of IgG antibodies against individual antigens of *Toxoplasma gondii* using immune blotting (Western blot) was developed and showed a high sensitivity of 98.51–100% and a high specificity of 99.5–100% [19]. This last study was conducted with the first international WHO standard anti-*Toxoplasma* serum (IgG), human, lyophilised, 20 IU/ampoule. Here, we demonstrated and validated Western blotting as a diagnostic alternative used as a confirmatory test compared to other methods to diagnose *Toxoplasma* infection and can be used in humans and cats [17,18,19]. 

The seroprevalence was comparable with other cities in China (11.7%) [20] but was higher than that in cities in Japan (6.7%) and Korea (2.2%) [21,22]. Compared to other reports from Mexico, the prevalence found in this study was low [10,11,12,13]. However, a survey from Izmir, Turkey, found that, in healthy stray cats, the seropositivity rates were 33.4% (342/1021) and 34.4% (352/1021) according to IFA and in-house ELISA, respectively, and further found a difference in the prevalence according to geographic zones of Izmir [23]. In a study conducted in Jalisco, 24 serum samples from cats were tested for anti-*Toxoplasma* antibodies through ELISA and were positive for IgG (70.8 and 8.3%). In this study, 297 serum samples of cats were tested by Western blotting, of which 14.8% were positive. The difference in this prevalence could be due to the number of models and the methodology used. However, using the Western blot method, a survey in Korea found a prevalence of 13.1%, similar to our results [24]. Another possibility is that, in the last 20 years, public policies have changed about the responsibility of owners with their pets and associations spread safe practices on food and care to reduce infections in their cats.

The frequency of infected cats was higher in municipalities with a high population density and according to the Jalisco government [14]: Zapopan has 9721.36 inhabitants per km^2^, Tlajomulco de Zúñiga 2459.97/km^2^, and Guadalajara 491.57/km^2^. However, in Tonalá, which has a high population of 4483.28 inhabitants per km^2^, infected cats (7.7%) were very close to San Pedro Tlaquepaque, with 862.68/km^2^ inhabitants, having the lowest frequency (5%) of positive cats. This may be due to the small number of samples analysed in the municipalities of Tonalá and Tlaquepaque. Conversely, a study in Turkey found that the prevalence of infection depends on the geographic characteristics of each region in one country [23].

Regarding age, our study found that cats one year or older were more likely to be seropositive, with a statistical difference of *p* < 0.001 (OR =3.9, 95%CI = 1.844 to 8.362). A similar study of cats from Angola reported that the odds of a cat being seropositive for *T. gondii* increases with age, considering an average factor of 1.58 for each 1-year increase in age, with other studies confirming this risk [10,25,26,27,28,29].

In addition to other attributable aspects, such as the amount of care taken by the owners of these cats regarding their diet, it is essential to consider that cats older than one year have a high risk of oocyst excretion and contamination for humans and other animals [30,31,32,33,34]. *T. gondii* infection was not significantly associated with whether cats had outdoor access, with positivity rates of 13.1 and 16.5% for those that did and did not, respectively. However, other studies in Latvia and Estonia demonstrated that cats with outdoor access were at higher risk of *T. gondii* infection, and outdoor access in cats is considered a risk factor [28,29]. This may be linked to the higher consumption of animals infected with *Toxoplasma gondii,* exposure to ground contaminated with faecal oocysts, humidity, and temperature. The coexistence of cats with other animals increases the risk of *Toxoplasma* infection 3.3 times fold. This result agrees with a study in which 90% of the cat positives analysed coexisted with dogs [33]. In contrast, another report showed the highest *Toxoplasma* infection rate in cats living in a group of more than one cat [13].

A national human survey determined that the state of Jalisco ranked 9th out of 32 in terms of states with high rates of seroprevalence [34]. However, the prevalence of a more current study found a decrease in the last sixty years [6]. The low prevalence of cats in our region can be this prevalence in humans. However, a cat infected with the parasite can release from 3 to 810 million oocysts for the first time, causing environmental contamination [3].

In addition, in a recent study of cats from Iran, the presence of oocysts in the faeces of cats was relatively low [35]. In one study, the excretion of *T. gondii* oocysts in feral cats in Korea was observed in 5/563 (0.89%) of cats studied [22]. These oocysts are highly resistant due to them surviving for a long time, especially in regions with hot and humid weather [36]. The metropolitan region of Guadalajara is characterised by warm, sub-humid weather, where the annual average temperature is 25.5 °C (ranging from 15 °C in winter to 37 °C in summer), with 850 mm of rainfall. These conditions allow for the maintenance and dissemination of oocysts, increasing the risk of transmission and accidental infection [36,37].

Toxoplasmosis is a problem of public health. The felids are the host that carries out the sexual cycle and can excrete the environmentally resistant oocysts in faeces. Cats can excrete millions of oocysts and a single cat can spread the infection to many hosts. For this reason, the diagnosis, treatment, and prevention of *Toxoplasma* infection in these pets are important [37,38]. Further studies in additional areas will be necessary to know the epidemiological status of toxoplasmosis cats in western Mexico.

## 4. Conclusions

The prevalence of *T. gondii* infection in cats in the metropolitan area of Guadalajara, Jalisco, was lower than two decades prior.

Public politics and the actions of associations to animal protection helped to decrease the higher prevalence of *Toxoplasma* infection in cats in the last two decades.

The Western blot is a good method to diagnose *Toxoplasma* infection in cats.

These results could be critical to researchers, health workers, veterinarians, and public policies related to toxoplasmosis.

## 5. Materials and Methods

### 5.1. Samples

Considering a previous seroprevalence of 70% and a finite population of 100,000 cats, the Open Epi software version 3.03 was used to calculate the sample size using the following formula:

(1) $$n=deff\;\times \;\frac{Npq}{\begin{array}{c} \frac{d^{2}}{1.96}\left(N-1\right)+pq\; \end{array}}\;$$

where *deff* (design effect) = 1, *N* (population size) = 100,000, *p* (heterogeneity) = 70%, *q* (1 − *p*) = 30%, and *d* (margin of error expressed in percentage) = 5%.

The sample size obtained according to the formula was 290 cats. Pet cat owners and cat shelter caretakers were identified and contacted with the help of veterinarians located in the Guadalajara metropolitan area. Cat owners in both contexts were informed of the objectives of the study. After, 53 cat owners and 7 cat shelters agreed to participate and proceeded to answer the cat habit questionnaires prior to blood sampling. 

Finally, the study was completed with 297 cats, comprising both those who lived at home with their owners (221/74.41%) and cats officially living in a shelter (76/25.58%). Blood samples were obtained by puncturing the jugular vein and were deposited in tubes with and without anticoagulants. Subsequently, the serum was centrifuged at 2000 rpm for 10 min, and serum was separated and preserved at −20 °C.

### 5.2. Questionnaire

The questionnaire included questions to obtain age, sex, municipality, and clinical history information such as vaccination schedule, observed hunting habits, cohabitation with other animals, and body condition, which were answered by the cat owners or the people responsible for shelters. Veterinarians performed a medical examination and obtained their clinical histories in all cases. The statistical analysis involved Western blot results and the completed questionnaires on the 297 cats.

### 5.3. Toxoplasma gondii Antigen-Derived Preparation

Tachyzoites of the virulent RH strain of *Toxoplasma gondii* were used for the experiments. These were obtained via intraperitoneal (i.p.) passage in six-week-old Swiss Webster female mice. Mice were injected via i.p. with 1 × 10^5^ tachyzoites per mL, harvested 3–4 days from peritoneal exudates, washed, and frozen at −20 °C [39].

### 5.4. Western Blot Method

Lysed *Toxoplasma gondii* tachyzoites (100 µg of protein per line) were separated by electrophoresis on a 12% sodium dodecyl sulfate-polyacrylamide gel and transferred onto nitrocellulose membranes using standard techniques [40]. The membranes were blocked with 5% fat-free milk in PBS and prepared into 0.3-cm strips. Membranes were then incubated overnight with cat serum samples diluted 1:100. The strips were washed, and anti-cat HRP-conjugated IgG was added at a 1:1000 dilution and incubated for two hours at room temperature. After washing, the presence of reactive bands was revealed by adding a substrate/chromogen solution containing H_2_O_2_ and 3,3′-diaminobenzidine (Sigma Catalogue-Aldrich D5637). Two or more bands were considered positive, whereas the absence of bands was deemed a negative result, as reported previously. Controls, serum was obtained from positive and negative cats, previously tested samples from our laboratory with ELISA and Western blot.

### 5.5. Immunoassay ELISA Method

Each ELISA plate was sensitised overnight at 4 °C with 100 µL of antigen per well previously diluted in 1× carbonate buffer. After the plates were washed three times with PBS 1X/tween-20 0.1% and blocked with 1% BSA in PBS 1X, 100 µL of diluted 1/400 serum of each cat was added in duplicate and incubated at room temperature for 2 h. After incubation, the plates were washed three times, and 100 µL of horseradish peroxidase-conjugated anti-feline IgG secondary antibody (HRP) diluted 1:1000 was added to each well. Plates were incubated for 1 h at 37 °C, washed three times with wash buffer, and 100 µL of ABTS was added as a developer. After 20 min of incubation, the absorbance was determined using a spectrophotometre at a wavelength of 405 nm. Serum samples were used as a positive control because of a lack of feline positive serum, and negative serum samples were collected from strict indoor cats. Of the 44 WB-positive serum samples, 42 were ELISA positive, and of the 40 WB-negative samples, 3 exceeded the cut-off limit and were considered positive. Thus, this method yielded a sensitivity of 95% and specificity of 93%.

The Kappa analysis between Western Blot and ELISA results showed a Kappa value of 0.886 with a standard deviation of 0.049 with a 95% confidence interval [0.790 to 0.983]; given an almost perfect agreement between the two methods. 

### 5.6. DNA Samples

Genomic DNA was extracted from 200 µL of non-coagulated blood using the GF-1 blood DNA extraction kit (Vivantis, Malaysia), following the manufacturer’s instructions. In brief, 20 μL of proteinase K was added and mixed with each blood sample and incubated for 10 min at 65 °C. Subsequently, 200 µL of absolute ethanol was added, and the mixture was run through a silica column. Pieces were washed with wash buffer solutions and DNA was eluted with sterile water. The samples were quantified using a spectrophotometer and stored at −20°C. The sensitivity PCR method described by Jalal et al. was used in our study to diagnose *Toxoplasma* [24].

### 5.7. Molecular Detection of Toxoplasma gondii

The PCR assay was performed using specific oligonucleotides with the following sequence: Tg1-5′-AAAAATGTGGGAATGAAAGAG-3′ Tg2-5-ACGAATCAACGGAACTGTAAT-3′, which targets the 35-fold repetitive gene B1 of *T. gondii* and amplified a fragment of 470 bp approximately [7]. In addition, internal amplification was controlled using specific oligonucleotides with the sequence B1-5′-ACCACCAACTTCATCCACGTTCACC-3′and B2-5′-CTTCTGACAACTGTGTTCACTAGC-3′ for the housekeeping gene β-globin [35]. For the PCR reaction, each reaction tube contained 2.5 µL of a 10× PCR buffer plus 0.5 µL of 10 mM dNTPs and 1 µL (10 pmol/µL) of mixed Tg1/Tg2 primers, 1 µL (10 pmol/µL) of B1/B2 primers, 0.3 µL (1U) of Taq polymerase (Vivantis, Malaysia), 3 µL of genomic DNA, and 17 µL of ultrapure water, in a final volume of 25 µL.

The amplification conditions were as follows: initial denaturation at 95 °C for 10 min, followed by 35 cycles at 94 °C for 1 min, 52 °C for 30 s, and 72 °C for 1 min. The final extension step was performed at 72 °C for seven minutes. For every 20 samples, a positive amplification control containing genomic DNA from *T. gondii* was included. The reactions were loaded and separated on a 1% agarose gel. The PCR reactions were acceptable if a band corresponding to the internal control (β-globin) appeared in the gel. The B-globin gene was used as an internal control to verify the DNA quality and absence of PCR inhibitors. DNA obtained from *Toxoplasma gondii* cultures (tachyzoites of the RH strain) was included in all PCRs as a positive control.

The B1 gene amplicon were subjected to DNA sequencing in both strands using the oligonucleotides described by Jalal and coworkers. The sequencing process was made in an ABI PRISM 3100 sequencer (Applied Biosystems Waltham, MA, USA). Electropherograms were analysed using the software Unipro UGENE v38.1.for IOS 12.

### 5.8. Statistical Analysis

The SPSS (v. 20.0) packages (IBM, Los Angeles, CA, USA) was used to perform all statistical analyses; univariable analyses included age and sex. The differences in the majority to *Toxoplasma gondii* positives and negatives between categories (age groups, sexes) were evaluated using the chi-square test (X^2^) and Fisher’s exact test. Risk factors were calculated using the odds ratio (OR) by multivariable analyses of logistic regression and the probability of each OR and their 95% confidence intervals (95%CI). *p* < 0.05 was considered statistically significant.

### 5.9. Ethical Approval

This study was performed in strict accordance with the recommendations of the Official Mexican Standards NOM-067, NOM-033-ZOO-1995, and NOM-062-ZOO-1999. The protocol was approved by the Biosafety, Research, and Ethics Committees of the University Center of Health Sciences of the University of Guadalajara, registration number CI-07619.

## 6. Limitations of the Work

The limitations of this study include a lack of information regarding the type of diet, consumption of undercooked meat, and cats living in shelters, because these cats can be less cared for than those who have a permanent home.

## Figures and Tables

**Table 1:** Distribution of anti-*Toxoplasma gondii* IgG antibodies by Western blot in cats from the metropolitan area of Guadalajara, Jalisco, Mexico.

	Negative		Positive		Total	*p*	OR	95%CI
	(*n* = 253)		(*n* = 44)		(*n* = 297)			
	No.	%	No.	%				
MUNICIPALITY						0.515		
Guadalajara	156	85.7	26	14.3	182	1.000	1.000	- - - - -
Tlajomulco	20	80.0	5	20.0	25	0.455	1.500	[0.517–4.348]
Tlaquepaque	19	95.0	1	5.0	20	0.271	0.316	[0.041–2.461]
Tonalá	12	92.3	1	7.7	13	0.514	0.500	[0.062–4.010]
Zapopan	46	80.7	11	19.3	57	0.363	1.435	[0.659–3.123]
Total	253	85.2	44	14.8	297			
AGE GROUP								
Under one year	119	92.2	10	7.8	129	1.000	1.000	- - - - -
One year and more	100	75.2	33	24.8	133	*p* < 0.001 *	3.927	[1.844–8.362]
Total	219	83.6	43	16.4	262			
SEX						0.452		
Female	128	83.7	25	16.3	153	1.000	1.000	- - - - -
Male	120	86.3	19	13.7	139	0.525	0.811	[0.425–1.547]
Total	248	84.9	44	15.1	292			

95%CI = 95% confidence intervals, OR = risk factor * statistically significant difference.

**Table 2:** Condition of the cats at the time of diagnosis for *Toxoplasma* infection.

Variables	Negative		Positive		Total	*p*	OR	95% CI
	No.	%	No.	%				
**^1^ Other animals in the same habitat**						0.326		
Yes	18	84.5	42	15.5	19	1.000	1.000	- - - - -
No	229	94.7	1	5.3	271	0.251	3.301	[0.429–25.398]
Total	247	85.2	43	14.8	290			
**Consumption of raw meat**						0.777		
Yes	226	85.6	38	14.4	264	1.000	1.000	- - - - -
No	22	84.6	4	15.4	26	0.891	1.081	[0.353–3.312]
Total	248	85.5	42	14.5	290			
**^2^ Complete vaccination**						1.000		
No	236	85.2	41	14.8	277	1.000	1.000	- - - - -
Yes	11	84.6	2	15.4	13	0.954	1.047	[0.224– 4.895]
Total	247	85.2	43	14.8	290			
**^3^ Current deworming**						0.306		
No	101	87.8	14	12.2	115	1.000	1.000	- - - - -
Yes	141	83.4	28	16.6	169	0.308	1.433	[0.718–2.858]
Total	242	85.2	42	14.8	284			
**^4^ Body condition**						0.330		
Good	179	84.4	33	15.6	212	1.000	1.000	- - - - -
Bad	72	88.9	9	11.1	81	0.333	0.678	[0.309–1.488]
Total	251	85.7	42	14.3	293			
**Outdoor access**						0.403		
Yes	133	86.9	20	13.1	153	0.404	1.000	[0.396–1.451]
No	116	83.5	23	16.5	139	1.000		

^1^ Refers to interaction with dogs, cats, or both in the same place. ^2^ Complete vaccination refers to current vaccination status against rabies, feline leukemia, feline viral rhinotracheitis, Feline calicivirus, and Feline panleukopenia virus. **^3^** Refers to cats that have received treatment against parasites. 95%CI = 95% confidence intervals, OR = risk factor in the last 6 months. ^4^ A regular body condition refers to cats with optimal weight, without evidence of inflammation in lymphoid nodes, no visible lesions, and pink mucosa in the mouth.

## Supplementary Materials

The following supporting information can be downloaded at: https://www.mdpi.com/article/10.3390/pathogens11010109/s1; Supplementary File S1: Confirmation by DNA sequencing of the B1 gene of *Toxoplasma gondii*.
