# Supplementary material for: Prevalence of Toxoplasma gondii Measured by Western Blot, ELISA and DNA Analysis, by PCR, in Cats of Western Mexico
Source: Pathogens. 2022 Jan 17;11(1):109. doi: 10.3390/pathogens11010109 (PMC8778430; doi:10.3390/pathogens11010109)
Supplement: Supplementary file 1 [file pathogens-11-00109-s001.zip › pathogens-1530745-supplementary.pdf]

Supplementary information.

Confirmation by DNA sequencing of the *B1* gene of *Toxoplasma gondii*.

Sample 21/10-222.

AAAAATGTGGGAATGAAAGAGACGCTAATGTGTTTGCATAGGTTGCAGTCACTGACGAGCT
CCCCTCTGCTGGCGAAAAGTGAAATTCATGAGTATCTGTGCAACTTTGGTGTATTCGCAGAT
TGGTCGCCTGCTATCGATAGTTGACCACGAACGCTTTAAAGCACAGGAGAAGAAGATCGTG
AAAGAATACGAGAAGAGGTACACAGAGATAGAAGTCGCTGTGGAGACAGCGAAGACTGC
GGGTGACTTCACTCCCGTCGCACCTGCAGCAGAGGAGTGCCGGGCAAGAAAATGAGATGC
CTAGAGGAGACACAGCGTGTTATGAACAAATCTATTGAGGTTTCGCGAAGAGGAGGGAAC
ATATTATATACAGAAGAAGAACAAGAGACGTGCCGCATGTCGCTAAGCCATCGGAAGGGA
TGCTCAGAAAATGGCACAGTATCACATTACAGTTCCGTTGATTCGT

Sample 24/10-223

AAAAATGTGGGAATGAAAGAGACGCTAATGTGTTTGCATAGGTTGCAGTCACTGACGAGCT
CCCCTCTGCTGGCGAAAAGTGAAATTCATGAGTATCTGTGCAACTTTGGTGTATTCGCAGAT
TGGTCGCCTGCTATCGATAGTTGACCACGTACGCTTTAAAGCACAGGAGAAGAAGATCGTC
AAAGAATACGAGAAGAGGTACACAGAGATAGAAGTCGCTGTGGAGACAGCGAAGACTGC
GGGTGACTTCACTCCCGTCGCACCTGCAGCAGAGGAGTGCCGGGCAAGAAAATGATATGC
CTAGAGGAGACACAGCGTGTTATGAACAATTCTATTGAGGTTTCGCGAAGAGGAGGGAAC
ATATTATATACAGAAGAAGAACAAGAGACGTGCCGCATGTCGCTAAGCCATCGGAAGGGA
TGCTCAGAAAATGGCACAGTATCACATTACAGTTCCGTTGATTCGT
